# Food security: contributions from science to a new and greener revolution

**DOI:** 10.1098/rstb.2009.0201

**Published:** 2010-01-12

**Authors:** John Beddington

**Affiliations:** Government Office for Science, Kingsgate House, 66–74 Victoria Street, London SW1E 6SW, UK

**Keywords:** food security, agriculture, climate change, energy, water, population

## Abstract

There is an intrinsic link between the challenge we face to ensure *food security* through the twenty-first century and other global issues, most notably *climate* change, *population growth* and the need to sustainably manage the world's rapidly growing demand for *energy* and *water*. Our progress in reducing global poverty and achieving the Millennium Development Goals will be determined to a great extent by how coherently these long-term challenges are tackled. A key question is whether we can feed a future nine billion people equitably, healthily and sustainably.

Science and technology can make a major contribution, by providing practical solutions. Securing this contribution requires that high priority be attached both to research and to facilitating the real world deployment of existing and emergent technologies. Put simply, we need a new, ‘green*er* revolution’. Important areas for focus include: crop improvement; smarter use of water and fertilizers; new pesticides and their effective management to avoid resistance problems; introduction of novel non-chemical approaches to crop protection; reduction of post-harvest losses; and more sustainable livestock and marine production. Techniques and technologies from many disciplines, ranging from biotechnology and engineering to newer fields such as nanotechnology, will be needed.

## 1. Introduction

After 20 years of low food commodity prices, the price shock of 2007/2008 brought agriculture, food production and food security sharply back into the limelight. Wheat and maize prices peaked at around triple their early 2005 levels, with an even higher peak in rice prices (IMF 2008). High commodity prices quickly fed into increased costs to consumers in developed and developing countries alike (FAO 2008*b*), escalating into civil unrest in some, ranging from strikes in Italy to riots in Haiti. The FAO estimated that the number of undernourished people in the world increased by 75 million in 2007, mainly attributed to high food prices. This brought the proportion of people in the world without access to sufficient food back to the levels of a decade ago (FAO 2008*c*). Commentators from the World Bank, FAO, USDA and the World Food Programme predicted further increases in the number of undernourished people in 2008, though the true figures may not become clear for some time (World Bank 2008*b*; Shapouri *et al.* 2009). Even before the price spike, the FAO estimated that more than 850 million people globally were undernourished, the great majority in developing countries (FAO Statistics Division 2006).

## 2. Short-term factors

A number of commentators have assessed the reasons behind the rapid price rises that reached their peak in 2008 (Abbott *et al*. 2008; Mitchell 2008; OECD/FAO 2008; Trostle 2008; Khan & Zaks 2009; Piesse & Thirtle 2009; World Bank 2009). There is now much agreement that short-term factors were dominant, although rather less on the weighting to be attached to each of these. The factors fall broadly into three categories: (i) those related to the fundamentals of supply and demand, (ii) government policy responses, and (iii) market/investment developments. To illustrate:

- *Supply and demand*: Poor harvests due to adverse weather conditions in regions such as Australia and the EU reduced the supply of cereals, particularly for wheat, and contributed to low world stocks (FAO 2008*a*). At the same time, the increased use of crops for biofuel production increased demand for maize (Mitchell 2008; Trostle 2008).
- *Policies*: Government policies in many countries have been successful in reducing stocks from arguably excessive 1980s levels, but have reduced the capacity to respond to speculation. By 2008, global stocks had declined to a level not seen for more than 30 years. At the same time, policy responses such as export bans and other trade restrictions from some countries facing food security crises exacerbated the problems, especially in the case of rice (Mitchell 2008).
- *Markets and investments*: A relatively new phenomenon is the extent to which food prices have been influenced by developments in non-food markets. Price transmission across commodities markets is illustrated by the correlation between energy and food prices (Goldman Sachs 2008; Piesse & Thirtle 2009). Linked to this is the role of large-scale index fund investment in commodities markets and speculation (Gilbert 2008; Levin & Coburn 2009). The depreciation of the US dollar in early 2008 has also been highlighted as an important factor by some (Abbott *et al*. 2008).

## 3. Long-term challenges

A number of important longer term trends and issues are also putting pressure on food production, in particular population increases, rising living standards, growing demand for energy, land and water, and climate change. While the contribution of these to the 2007/2008 price peak has been debated (Mitchell 2008; Trostle 2008; Piesse & Thirtle 2009), these long-run factors will ultimately present a major and increasing challenge to global food security. They are also intimately linked and will impact across societies in areas such as poverty alleviation and regional security, as well as exerting pressure on food production.

### (a) Population increase and urbanization

Global population is set to increase to around nine billion by mid-century, rising at a rate of six million people per month, with Africa's population alone projected to double from one billion to two billion (UNPD 2006). This continued population increase combines with other transformational change, particularly in the developing world as people move from rural livelihoods to cities, cities that will need to be serviced with food, water and energy. Half the world's population now live in cities, a figure projected to rise to 60 per cent by 2030 (UNPD 2007). It is estimated that there will be 26 cities with greater than 10 million inhabitants in 2025, up from 19 today. Five of these new ‘megacities’ will be in Asia (UN-HABITAT 2008).

### (b) Economic changes leading to changes in demand for food

Population increase will be coupled to increasing prosperity. Economic advances projected for the developing world will help lift millions from poverty, but in other respects will add to the challenges. As incomes rise in developing and middle-income countries, people eat more meat and dairy products, causing a rapid growth in demand for agricultural commodities to feed livestock. Strong growth in demand over the past few decades has been driven particularly by rising consumption in China and Brazil, and the future trend is likely to be strongly influenced also by the extent of income growth in India and sub-Saharan Africa, where *per capita* meat consumption is still low (FAO 2003).

The FAO projects total crop and livestock demand and production will rise by around 40 per cent between 2009 and 2030, i.e. a yearly increase of 1.5 per cent. However, this overall figure conceals the larger increase in meat demand (FAO 2006*a*; UNPD 2006). The World Bank (2008*a*) predicts a 50 per cent rise in cereals demand compared with an 85 per cent increase for meat between 2000 and 2030. Other assessments predict a doubling of meat demand by 2050 (Beintema *et al*. 2008). The overall projected rate of demand growth is lower than in previous decades (FAO 2006*a*; IPCC 2007), but must be met within the greater constraints on land, water and energy use outlined below.

### (c) Rising demand for energy, water and land

**Figure 1.**
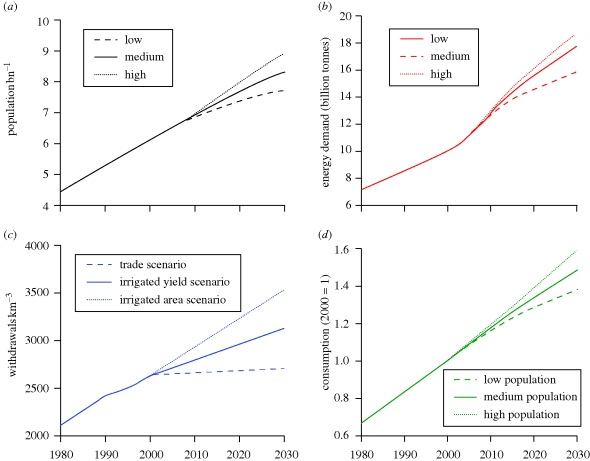
(*a*) Global population (1980–2030, from UNPD 2006). (*b*) Global primary energy demand in billion tonnes oil equivalent (1980–2030, from IEA 2007). (*c*) Global withdrawals of water for agriculture (1980–2030, from de Fraiture *et al.* 2007). (*d*) Global food consumption, from FAO (2006*b*) and UNPD (2006).

Projections for population, energy, water and food are presented in figure 1 and discussed in more detail in the following sections.

#### (i) Energy

Energy demand is projected to increase by 45 per cent between 2006 and 2030, based on the IEA's (2008) reference scenario. Biofuels for transport and biomass for heat and electricity will be used to meet some of this demand, leading to greater competition for land and crops between energy and food markets (Mitchell 2008). In addition, the production of the mineral fertilizers on which modern, intensive agriculture has come to rely to replenish nutrient-depleted soils requires significant energy. The up to fivefold increase in fertilizer prices between 2005 and 2008 was strongly influenced by the soaring oil price during this period (Piesse & Thirtle 2009), alongside production capacity constraints linked to the availability of phosphates, potash and other mineral ores.

#### (ii) Water

Today, 1.2 billion people live in areas already affected by water scarcity, and this figure is projected to increase as global water demand rises (Molden *et al.* 2007). Water demand is a function of population, incomes, diets and the extent of irrigated agriculture, leading to a wide range of projections into the 2020s and 2050s (Shiklomanov 2000; de Fraiture *et al.* 2007; Shen *et al.* 2008). It has been estimated that crop water consumption could increase by 70–90% by 2050, in the absence of measures to mitigate this, while modelling based on the IPCC's SRES scenarios suggests that total global water demand will rise by 35–60% between 2000 and 2025 (de Fraiture *et al.* 2007; Shen *et al.* 2008).

Agriculture is by far the largest user of water worldwide, at around 70 per cent of total supplies (FAO Global Perspective Studies Unit 2007). However, as economies develop and diversify, agriculture declines in significance as a contributor to gross domestic product (GDP) and as one reflection of this in many developed countries it has ceased to be the dominant user of water. As a consequence, agriculture's share of total water demand is projected to drop to under 60 per cent by 2050 (de Fraiture *et al.* 2007). For those developing economies still centred on agriculture, future transitions will see industry and housing put increasing pressure on agriculture's share of water, increasing competition between sectors and heightening the risk of over-extraction of groundwater. Poorly managed irrigation schemes have already led to widespread problems with salinity and waterlogging, affecting, for example, 25 per cent of the irrigated land in Pakistan (World Bank 2006; Hazell & Wood 2008). In some developed countries too, lack of water regularly limits crop production; Australia being the prime example of the past few years.

Over-extraction in cities also brings enhanced risk of anthropogenic subsidence, exacerbating future flood risks already set to rise through climate change impacts and socio-economic changes (Nicholls *et al*. 2007).

At the same time, there is considerable potential to increase food production in those parts of sub-Saharan Africa where water availability is fundamentally not a problem, in particular by expanding irrigated farming systems (Markwei *et al*. 2008).

The relatively recent concept of ‘virtual’ or ‘embedded’ water is used to measure the amount of water used to produce foodstuffs (as well as other commodities). Estimates suggest that exported foods account for around 16–26% of the total water used for food production worldwide, suggesting significant potential for more efficient global use of water via trade (Zimmer & Renault 2003; Hoekstra & Chapagain 2007). The ideal would be for water-scarce countries to import water-intensive goods from those with an abundance of water. In practice, however, virtual water use is more often a product of trade than a driver of trade, leading to poor correlation between the water resources of a country and the amount of virtual water imported or exported (Hoekstra & Chapagain 2008).

There are similarities here with the concept of ‘global commons’ for food production. This is based on the assessment that, particularly as climate change advances, some world regions such as Western and Central Europe and the northeastern US states will dominate as the most efficient areas for agriculture (Muller *et al*. 2006).

#### (iii) Land

Around 1600 million hectares of land are currently cultivated for crops (FAO Statistics Division 2008). The FAO estimates that, ignoring impacts on biodiversity and the carbon cycle, some 2400 million hectares of land globally would be at least moderately suitable for wheat, rice and grain maize cultivation, around 18 per cent of the total world land area (FAO/IIASA 2000). Other studies have variously suggested between 50 and 1600 million hectares of land to be suitable for agricultural expansion (EEA 2007; de Fraiture *et al.* 2007; CE Delft 2008). The fact that estimates range so widely reflects the major uncertainties involved in assessing the potential of land for agricultural production on a global scale. Indeed, estimates even of current land usage are diverse (de Fraiture *et al.* 2007; FAO Statistics Division 2008). It seems inevitable, however, that demand for land will progressively increase, both for food production and linked to the urbanization and energy trends noted above (de Fraiture *et al.* 2007). This growing competition can be illustrated by increased purchases and leases of land in Africa, Latin America, Central Asia and southeast Asia by a range of investors such as multinational companies and countries with limited domestic production potential and/or large Sovereign Wealth Funds (Cotula *et al*. 2009).

Perhaps the most important competitor for land is the natural environment itself. Sustainable development and agriculture require safeguarding ecosystems and biodiversity. Forests are also among the areas particularly important from a climate change perspective, given their key role in storing carbon. The substantial increases in food production required will thus need to fit with efforts to reduce deforestation.

### (d) Climate change

The pernicious backdrop against which these demands must be met is one of rising global temperatures, impacting on water, food and ecosystems in all regions, and with extreme weather events set to become both more severe and more frequent (IPCC 2007). The need both to mitigate climate change and to adapt to that which it is too late already to avoid is clear. Global greenhouse gas emissions must be reduced by at least 50–60% by 2050 compared with current levels (IPCC 2007). The UK set an important political lead by committing to an 80 per cent domestic reduction by 2050 (Climate Change Act 2008).

The substantial increase in food production that will be required to meet global demand must therefore be achieved in parallel to delivering a steep reduction in greenhouse gas emissions. Agriculture is a major contributor to climate change, responsible for around 10–12% of emissions, even excluding the impact of deforestation (IPCC 2007). Yet, the scope to reduce emissions from agriculture and specific mechanisms and technologies for this are relatively unexplored, in comparison, for example, with energy generation and transport, and are important areas for future research and innovation. One assessment has suggested a realistic abatement potential in the UK of up to 11 MtCO2e by 2020, or about a quarter of current emissions from UK agriculture (Committee on Climate Change 2008).

These gains could be delivered through a combination of changing farming practices and employing new technologies, in particular focussing on better use of fertilizers, breeding programmes for crops and livestock, and improvements in drainage. Similar analysis at a global level suggests technical abatement potential of up to 60 per cent of sectoral emissions, with a carbon price up to €60/tonne. The realization of such potential will depend on the policies adopted by governments to incentivize change (Khan & Zaks 2009).

There is some evidence that global average yields could rise with a small temperature increase (1–3°C) (IPCC 2007). However, this conceals large regional variations. In the already hot tropics, mainly populated by developing countries, any increase in temperature is likely to be detrimental to food production (Cline 2007). Rising sea levels and flooding will hit hardest in the megadeltas, which are important areas of cultivation, as well as population centres, and will impact too on water quality for many (IPCC 2007).

Sub-Saharan Africa is likely to experience some of the worst impacts, losing up to 9 per cent of potential agricultural land by 2080, and has one of the lowest capacities to adapt (Fischer *et al*. 2002; Lobell *et al*. 2008). The area of semi-arid and arid land in Africa could increase by 5–8% by the 2080s, with wheat production disappearing from Africa on this time scale (Fischer *et al*. 2005). For an already drought-prone continent, climate change impacts will bring an even greater risk to food production in many areas (FAO 2005).

Rice production in Asia is also likely to be hard hit by climate change, as rice is particularly vulnerable to high temperatures (IPCC 2007). Also impacting on food production in Asia is the potential loss of dry-season Himalayan glacial meltwater, on which hundreds of millions of people in the Indian sub-continent and China are dependent. These glaciers are expected to lose 80 per cent of their volume by 2035 (Stern 2006; IPCC 2007; Xu *et al*. 2009).

Even in temperate regions, farmers will need to adapt to changing temperature and rainfall patterns, and the increased likelihood of extreme events such as floods and droughts. The impact of changing temperatures on the range of pests and diseases is uncertain, but this too cannot be ignored (Stireman *et al*. 2005; OSI 2006; Diffenbaugh *et al*. 2008).

### (e) Other environmental factors

The substantial increase in food production required will also have to be achieved within other environmental constraints, including maintenance of soil and water quality and biodiversity conservation. Past farming practices have caused soil degradation both in developed countries such as the UK and in the developing world.

The problem is particularly severe in West Africa, where soils are inherently low in organic carbon, limiting nutrient and water capacity/uptake, and annual losses of soil organic carbon may average 2–5% with continuous cultivation (Bationo *et al.* 2007). The International Center for Soil Fertility and Agricultural Development estimates that Africa loses eight million tonnes of soil nutrients per year, and over 95 million hectares of land have been degraded to the point of greatly reduced productivity (Henao & Baanante 2006). Retaining soil fertility may be one of the major challenges in the development of agriculture in this region.

The intensification of agriculture also risks degrading water resources and impacting farmland and aquatic ecosystems through the excessive or untargeted use of fertilizers and pesticides. It has been predicted that a doubling of food production between 2000 and 2050 could be associated with two to three times more eutrophication of marine and freshwater ecosystems, driven by increased levels of nitrogen and phosphorus (Tilman *et al*. 2001). Agriculture has been cited as one factor behind the most recent high rate of species extinction, which for the past few hundred years has been as much as 1000 times the background rate (MA 2005).

However, it is important to note too that the intensification of agriculture and modern agricultural practices have brought substantial environmental benefits. For example, while cereal production in Asia doubled between 1970 and 1995, the total land area cultivated with cereals increased by only 4 per cent (IFPRI 2002).

Had global cereal yields in 2004 remained at 1961 levels, 1.4 billion hectares of additional land of equivalent quality would need to have been found to match production levels achieved. Even had the land been available, the environmental consequences of such an extensification would have been devastating (Cassman *et al*. 2005).

## 4. The contribution of science and technnology

The challenge is clear. The world must produce 40 per cent more food, with limited land and water, using less energy, fertilizer and pesticide—by 2030—at the same time as bringing down sharply the level of greenhouse gases emitted globally, and while coping with the impact of climate changes that cannot be avoided. To do so, we must maximize both the use of those technologies already developed and generate and exploit new scientific discoveries. We need a new and green*er* revolution, a revolution with science and technology at its heart.

History demonstrates science and technology can provide huge increases in yield growth. Cereal yields in East Asia rose by 2.8 per cent a year between 1961 and 2004 (World Bank 2008*a*), or over three times in total over the period, enabled by modern farming practices, including irrigation, use of fertilizers and pesticides and the development of new more productive crop varieties (Evenson & Gollin 2003). The contrast with Africa is marked, where the failure of such approaches to take hold has contributed to a stagnation in yields that has endured for several decades (Markwei *et al*. 2008). Yet, the potential exists in sub-Saharan Africa for a step change in productivity. Average yields of maize are around 1 tonne ha^−1^ while demonstration farms routinely achieve yields of 3–6 tonnes ha^−1^ (World Bank 2008*a*).

**Figure 2.**
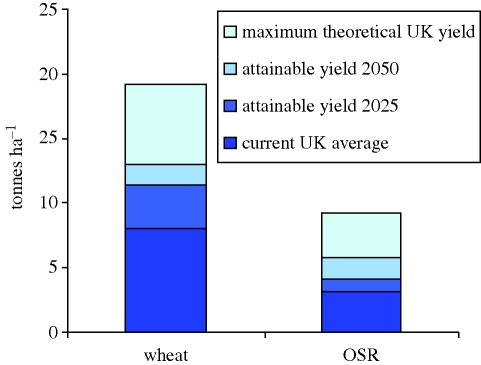
The theoretical and obtainable potential yields for wheat and oilseed rape (OSR) in the UK (from Berry & Spink 2006; Sylvester-Bradley *et al*. 2006; Street *et al*. 2009).

There is great potential even in developed countries such as the UK for agriculture to deliver substantially increased productivity (Street *et al*. 2009). It has been estimated (figure 2) that the theoretical yield potentials of wheat and oilseed rape in the UK are 19.2 and 9.2 tonnes ha^−1^, respectively (Berry & Spink 2006; Sylvester-Bradley *et al*. 2006). Average UK farms currently deliver around 7.7 and 3.2 tonnes ha^−1^ (Street *et al*. 2009). Realizing even a fraction of this higher potential in the UK and similar climates could have a substantial impact on global production—the UK alone produced 3 per cent of the world's wheat in 2008 (FAO 2008*b*).

There are substantial constraints to releasing this potential, and implications for water and nitrogen use and soil quality. But these figures illustrate that, though yields have begun to plateau, with growth in global aggregate yield averaging 1.1 per cent between 1990 and 2007, down from 2.0 per cent from 1970–1990 (Trostle 2008), this is unlikely to be due to fundamental limits.

A multitude of approaches and technologies have the potential to contribute to achieving the long-term goal of sustainable food security (Markwei *et al*. 2008; National Academy of Sciences 2008; Pretty 2008; Evans 2009; Royal Society 2009; Royal Society of Chemistry 2009). As noted, even with existing technologies, there is the opportunity for great gains to be made. In this respect, socio-economic systems and policies will often be key to ensuring the benefits of technologies that can be fully exploited, particularly in regions such as sub-Saharan Africa. The differing cultural, economic and agro-ecological conditions between countries will mean that there can be no ‘one size fits all’ solution and tailored approaches to, for example, financing will be important (Markwei *et al*. 2008; World Bank 2008*a*; Royal Society 2009). Further critical issues in developing countries include the development of rural infrastructure and improved extension services, as well as the availability of inputs such as fertilizers and pesticides and mechanisms by which local farmers can access markets.

At the same time, the benefit of investment in agricultural research is clear, with one study estimating an average internal rate of return of 43 per cent in 700 R&D projects evaluated in developing countries. Similar rates of return have also been found for agricultural research in developed countries (Alston *et al*. 2000).

Yet, developed country spending on public agricultural R&D has seen an extended period of stagnation, and while private sector research has grown, its commercial orientation has placed emphasis on cost reduction rather than yield increases (Pardey 2006; Trostle 2008). In particular, there has been less incentive to address the needs of poorer farmers and to focus on more strategic, longer term issues—93 per cent of private agricultural R&D takes place in developed countries. Despite the increase in private sector R&D, the *intensity* of global spend (the percentage of agricultural GDP spent on research) declined slightly in the last decade of the twentieth century (Pardey 2006).

The trend in developing country research has been highly variable by region, including relatively strong growth in Asia. Notable, however, has been the decline in the intensity of research in sub-Saharan Africa, despite commitments to the contrary in the Maputo Declaration. The failure of the green revolution to take hold in Africa has been partly attributed to a lack of development of African scientific, technological and entrepreneurial capacity to exploit international advances in agricultural science, for example to adapt new varieties of crops to specific regions (Evenson & Gollin 2003; Juma 2008). It is therefore a positive development that evidence is emerging that consistent agricultural growth is finally starting to be realized across the region as a whole (Badiane 2008; World Bank 2008*a*).

## 5. Solutions from science and technology

Highlighted below are some of the major areas where future research and innovation have the potential to pay dividends.

### (a) Crop improvement

Crop improvement through breeding has been key to the past successes of agriculture. Much of the growth in major crop yields in developing countries (21% between 1961 and 1980 and 50% between 1981 and 2000) has been attributed to the use of improved crop varieties (Evenson & Gollin 2003). New varieties can present a win–win for yields and environmental impact, especially if the focus is on improving the resource use efficiency of crops. For instance, conventional breeding using selection for transpiration efficiency has been used to develop drought-resistant wheat (Rebetzke *et al*. 2002).

The introduction of improved crop varieties has been most successful for the major crops in favourable agro-ecological areas (Evenson & Gollin 2003). While there have also been successes in less favoured areas and for minor crops, for example cassava, there is a need for enhanced research particularly for the subsistence crops of sub-Saharan Africa, such as sorghum and millet. Research to develop improved crops that are appropriate for local conditions is vital to their widespread adoption, and schemes to promote greater participation of local farmers in research have accelerated both development and adoption (Walker 2007; Somado *et al*. 2008).

New genomic techniques, such as marker assisted breeding, allow greater selectivity and reduce the element of chance in plant breeding. These techniques have been used to promote a range of qualities such as submergence tolerance in rice and increased resistance to pests and diseases (Collard & Mackill 2008). Successes to date using genomics have included the development of more disease-resistant cassava, now distributed to smallholders in Burundi, the Democratic Republic of Congo, Rwanda and Uganda (Okogbenin *et al*. 2007). Looking ahead, genomic techniques have strong potential as one of the key technologies to offer solutions, accelerating our ability to develop varieties with characteristics of drought, heat and saline resistance, as well as resistance to pests and disease, although environmental and food safety conditions will need to be met (Møller *et al*. 2009).

A current challenge is the re-emergence of stem rust as a major threat to global wheat production, with a new variety identified in Uganda in 1999, dubbed Ug99 (Pretorius *et al*. 2000). Stem rust has historically been one of the most damaging and widespread of wheat diseases, controlled for decades by international collaboration to breed for genetic resistance and through a programme of cultivar releases. The re-emergence of stem rust now threatens 20 per cent of the world's wheat in Central and North Africa, the Middle East and Asia. One study suggested potential losses in these areas of 9–60 million tonnes, depending on the scenario used, in the region of 1–10% of global wheat production (Hodson *et al*. 2005; Singh *et al*. 2006).

This is now being addressed through the Global Rust Initiative, a major research initiative under the umbrella of the International Maize and Wheat Improvement Center (CIMMYT) and the International Center for Agricultural Research in Dry Areas (ICARDA), which has led to new resistant, high yielding strains being identified, which are now being distributed globally.

Biofortification through crop breeding and changes in production to add micro-nutrients to staple crops may become increasingly important as means to address malnutrition in developing countries. The World Health Organization estimates that vitamin A deficiency alone is responsible for causing blindness in up to half a million children each year. Increased levels of nutrients including vitamin A, iron and zinc have already been demonstrated in staples such as rice and sweet potato (Nestel *et al*. 2006; Gilani & Nasim 2007).

### (b) Crop protection

Crops are attacked by a great variety of pests, diseases and weeds. A key challenge to the protection of current production is the emergence of new pests and diseases, in addition to the spread of current diseases (OSI 2006). The growing, rapid global movement of people and agricultural materials has brought a constant stream of new crop diseases and pests, and allowed more rapid mixing and evolution of virulent new disease strains, such as Ug99 referred to above (Levine & D'Antonio 2003).

Crop protection through pesticides has made a significant contribution to growth in productivity since the 1950s. However, losses due to pests globally are still high. The figures vary between countries and crops, but one estimate suggests an overall loss of around 40 per cent (Yudelman *et al*. 1998). Another more recent assessment suggests losses of 26–29% for soyabean and wheat, and 30–40% for maize, rice and potatoes (Oerke 2006). The same study suggests that losses for wheat could be as high as 50 per cent without effective plant protection, and even higher for other crops.

Improved crop protection in the face of new pests and diseases, as well as resistant strains of current diseases, will rely on a variety of approaches. The well-managed use of conventional pesticides must continue to play a key role, set in the context of the major losses noted above. However, there are also opportunities for greater use of integrated pest management techniques, the stimulation of plants' natural defences and the use of ‘semiochemicals’ including insect pheromones to dissuade insects from attacking crops (Cook *et al*. 2007; Pickett *et al*. 2007; Khan *et al.* 2008; Hassanali *et al.* 2008).

Crop losses after harvesting are also significant. Most susceptible are fruits, vegetables and root crops, but cereal staples are also vulnerable. Losses arise from pests and diseases, physiological deterioration from high temperatures or low atmospheric humidity, and physical damage.

Solutions range from careful harvesting and packaging to more advanced storage technologies and use of pesticides and fumigants. The need to research new technologies and approaches has been given new impetus by the banning of the fumigant methyl bromide in many countries due to its ozone-depleting effects. For example, inert dusts to protect against insect storage pests have proved successful in southern and eastern Africa (Morris *et al.* 2006).

### (c) Sustainable livestock farming

Demand for meat is projected to increase by 85 per cent by 2030 (World Bank 2008*a*). In terms of energy conversion, the production of meat is an inherently inefficient process. Figures vary for the ratio of conversion of animal feed to meat, but recent figures are 1.8 : 1 for chicken and between 5 : 1 and 10 : 1 for beef, depending on the production system (Garnett 2008; Trostle 2008). Around a third of the global production of cereals is used for animal feed (FAO 2006*a*).

Livestock farming also makes a significant contribution to climate change. It has been estimated that the sector is currently responsible for around a quarter of global anthropogenic methane emissions, and 14 per cent of anthropogenic nitrous oxide emissions. Total methane and nitrous oxide emissions from livestock are expected to grow by 50–60% by 2030 (FAO 2003).

Although there are strong arguments, including that linked to health considerations in developed countries, for efforts to reduce demand for meat, it is clear that the new greener revolution will need to address livestock as well as crops. The same issues of genetic improvement, efficient use of resources and protection from disease need to be tackled.

Particular constraints to the genetic improvement of livestock are their genetic complexity, the relatively long life cycle of livestock, particularly cattle, and the need to protect animal welfare and genetic diversity. Advances in areas such as molecular genetics, genome sequencing and reproductive technologies, dubbed ‘precision animal breeding’ attempt to overcome these difficulties. Marker assisted selection has been used, for example, to increase the litter size of pigs. There is undoubtedly much potential left to be exploited, for example through the use of information from across the genome for selection, rather than just individual genes or sections of DNA (Flint & Woolliams 2008).

It has been noted that the genetic diversity in livestock in intensive production systems is low, and that diversity has been lost as farmers switch to ‘industrial’ breeds from native lines, particularly in developed countries. Efforts to maintain diversity will need a mix of solutions, including protection of rare breeds and wild relatives, ensuring the genetic diversity of industrial breeds through selection programmes and cryoconservation of genetic information in gene banks (Taberlet 2007; Flint & Woolliams 2008).

A key limitation to animal productivity is disease. The scale of cost of animal diseases is starkly highlighted by the impact of outbreaks such as the Foot and Mouth Disease epidemics in the UK in 2001 and 2007 and bovine spongiform encephalopathy, and bovine tuberculosis in Botswana in the mid-1990s. The overall impact of animal disease on the UK has been estimated at 17 per cent of production, compared with 35–50% in developing countries (Flint & Woolliams 2008).

Increasing global temperatures will extend the range of certain animal disease spreading vectors such as mosquitoes, ticks and midges, and could bring the threat of animal diseases such as African Horse Sickness and West Nile Virus to areas where they have not historically been present, such as northern Europe (Bayliss & Githeko 2006).

A priority in combating animal disease is the development of vaccines. There are great hopes that recent genome sequencing of strains of African swine fever may lead to a vaccine for this disease. African swine fever has caused severe losses to pig production in many African countries over the past decade. Research has also produced quick diagnostic tests for diseases such as rinderpest, a fatal disease primarily affecting cattle in the Somali ecosystem in eastern Africa (BBSRC 2005). Rapid diagnosis in conjunction with vaccination programmes has led to the almost complete eradication of the rinderpest virus, which would make it only the second virus ever to be eradicated, after smallpox in 1979 (Normile 2008).

### (d) Fisheries and aquaculture

Sustainable fisheries are a priority for global food security, with fish comprising half the dietary protein for 400 million people in the world's poorest countries, and a fifth of protein nutrition in developing countries as a whole. Both capture fisheries and, increasingly, aquaculture are important, with the latter averaging an annual growth of 8.7 per cent per year since 1970 (FAO 2006*b*,*d*). However, the significant threat to fisheries from over-fishing, climate change and ocean acidification is well acknowledged.

As is the case for land-based agriculture, maximizing the contribution of rivers, seas and oceans to sustainable food production will require both effective policies and the judicious use of technologies. Greater consideration of the impact of fishing at the ecosystem level, and not only on individual species, will be an important part of improving fisheries management (Pikitch *et al*. 2004; FAO 2008*d*). Many of the tools for better management, such as tested and enforceable harvest strategies and rights-based management, are already available but need to be implemented (Beddington *et al*. 2007).

Technological innovations are already making an important contribution to achieving both economic and environmental goals, for example in capture fisheries enabling greater selectivity in fishing gear to minimize harmful impacts on the environment (FAO 2006*b*).

While much of the technology used in aquaculture remains relatively simple, more advanced technologies are also being applied. For example, the WorldFish centre has developed genetically improved strains of Nile tilapia for on-farm production and extended these to farmers in six Asian countries. An assessment of on-farm trials showed yield gains of 78 per cent in Bangladesh, achieved with no increase in production costs, and an internal rate of return of 70 per cent taking account of the costs of both research and dissemination (Deb & Dey 2005).

### (e) Mechanization and engineering

Agricultural engineering and mechanization were key enablers for the rise of large-scale industrial agriculture, historically delivering the step changes in productivity per unit of manpower which allowed countries to transform from agriculture to industry-based economies.

Mechanization of agriculture in Brazil over the last decade has supported a doubling in grain production with only moderate increases in land use (Embrapa (Brazilian Agricultural Research Corporation) 2008, personal communication). In the UK and the EU, engineering solutions have been found to allow larger more efficient harvesting machines to be used without significantly increasing damage to the soil structure through compaction (Godwin *et al*. 2008).

The importance of engineering is further illustrated by estimates that only 50 per cent of the impact of crop protection products is accounted for by the effectiveness of the product itself; the rest is dependent on factors such as the timing of the application and the precision of delivery (Robinson 2008).

Increased ‘precision farming’ could have important benefits for the management and efficient use of resources in agriculture. It encompasses a range of technologies, including for information and communication, monitoring (e.g. remote sensing and global positioning systems (GPS)) and automated process control. For example, GPS systems allow accurate control of tractor position and movements, enabling the precise delivery of seed and other inputs. Tractor-based and remote (aerial or satellite) sensors can be used to determine soil and plant characteristics to enable early detection of disease and water stress (Mondal & Tewair 2007).

In contrast to these high-tech methods, low-tech and small-scale technologies such as drip irrigation and the technology for precision fertilizer application have been trialled successfully in the developing world. ‘Microdosing’ of millet and sorghum with fertilizers in Burkina Faso, Mali and Niger, while labour intensive, has increased yields by 44–120% using only small amounts of fertilizer (Tabo *et al*. 2007). The approach involves just a few grams of fertilizer being applied to the base of each plant.

### (f) Nanotechnologies

In the coming years, the maturing field of nanotechnology is likely to bring radical new products and approaches to assist crop production. Exploiting a convergence of nanotechnologies, genomics and micro-electronics, new diagnostic tools for animal and plant diseases are dramatically improving our capacity to detect and monitor the spread of plant and animal diseases (OSI 2006). Already today, nanotechnologies have delivered improvements to pesticide delivery through encapsulation and controlled release methods. Capsules can be inert until in contact with leaves or insect digestive tracts, at which point they release the pesticide (Green & Beestman 2007). In combination with the use of nanoemulsions, pesticides can be applied more easily and safely. Smart, nanotechnology-based, sensors, applied to the field, may in future allow early detection of disease and monitoring of soil conditions to improve application of water, fertilizers and pesticides (Joseph & Morrison 2006). However, as with any new technology, the potential risks must be investigated and weighed against the benefits.

## 6. Conclusions

Food security through the twenty-first century is achievable, but must be tackled coherently with other global challenges. The key questions for policy-makers and scientists are these:

- Can nine billion people be fed equitably, healthily and sustainably?
- Can we cope with the future demands on water?
- Can we provide enough energy to supply the growing population coming out of poverty?
- Can we do all this while mitigating and adapting to climate change?

These issues are inextricably linked. Science has contributed greatly in the past to finding solutions, and it can do so into the future if the investments are made. A new green*er* revolution can be built on the foundations of the first green revolution, but we will need to fully explore the range of science and technology opportunities at our disposal in the twenty-first century in order to overcome the greater constraints. This vital contribution from science will not happen by default.
